# NeurimmiRs and Postoperative Delirium in Elderly Patients Undergoing Total Hip/Knee Replacement: A Pilot Study

**DOI:** 10.3389/fnagi.2017.00200

**Published:** 2017-06-23

**Authors:** Rui Dong, Lingling Sun, Yayuan Lu, Xi Yang, Mian Peng, Zongze Zhang

**Affiliations:** Department of Anesthesiology, Zhongnan Hospital of Wuhan UniversityWuhan, China

**Keywords:** postoperative delirium, microRNA, neuroinflammation, surgery, elderly patients

## Abstract

**Objective:** Postoperative delirium (POD) is a frequent complication after surgery and its occurrence is associated with poor outcomes. The pathophysiology of this complication is not clear, but identification of risk factors is important for positive postoperative outcomes. The purpose of this study was to investigate the associations between the preoperative expression levels of microRNA (miR)-146a, miR-125b, and miR-181c in cerebrospinal fluid (CSF) and serum and the development and severity of POD.

**Methods:** Forty elderly patients aged 65 years old and older admitted for elective total hip/knee replacement under spinal anesthesia. Preoperatively, baseline cognitive function was assessed using the Mini-Mental State Examination. Each patient was interviewed daily on the first and second postoperative days. Delirium was diagnosed using the Confusion Assessment Method, and delirium severity was measured using the Memorial Delirium Assessment Scale (MDAS). Preoperative serum and CSF miR levels were determined by quantitative real-time PCR (qRT-PCR).

**Results:** POD was detected in 27.5% (11/40) of patients. Up-regulation of miR-146a and miR-181c in CSF and down-regulation of miR-146a in serum were observed preoperatively in patients who developed POD, while patients with and without POD did not differ in serum or CSF levels of miR-125b. Delirious patients had higher CSF/serum ratios of miR-146a and miR-181c levels than non-delirious patients. The lower CSF miR-146a and CSF/serum miR-146a ratios were significantly associated with milder POD severity, represented by a lower MDAS score.

**Conclusion:** The dysregulation of preoperative miR-146a and miR-181c in CSF and serum was associated with the development and severity of POD. These NeurimmiRs might participate in the neuropathogenesis of POD, pending further investigations.

**Clinical trial registration:** this study was registered at ClinicalTrials.gov (NCT02817386).

## Introduction

Postoperative delirium (POD), an acute, transient, fluctuating disturbance in attention, cognition, and level of consciousness, is a common (15–53%) postoperative complication (Marcantonio et al., 1994; Liu and Leung, 2000; Sieber and Barnett, 2011), and it is associated with longer hospital stays, worse functional outcomes, higher healthcare costs, and increased mortality (Shim and Leung, 2012). However, at the current time, effective prevention and treatment are not only hampered by lack of knowledge about the neuropathogenesis of POD but also by a lack of biomarker(s) that could identify the risk for the development of POD. Neuroinflammation, in particular, has frequently been cited as an important etiological factor associated with the development of POD (van Gool et al., 2010).

Neuroinflammation plays a crucial role in POD (Cerejeira et al., 2010; van Gool et al., 2010). Surgical trauma engages the innate immune system to release proinflammatory cytokines, in particular interleukin-1β (IL-1β) and tumor necrosis factor α (TNF-α). These cytokines signals can be transmitted to the brain and lead to neuroinflammation through direct neural pathways (via primary autonomic afferents), transport across the blood–brain barrier (BBB), or entry via the disrupted BBB. Increased brain proinflammatory cytokines can overactivate microglia, which induces further cytokine release in cerebral tissue and fuels a vicious cycle of neuroinflammation (Perry, 2004; Teeling and Perry, 2009). Furthermore, overactivated microglia creates a neurotoxic response, causes neuronal injury, and affects neuronal function, leading to POD (Garden and Moller, 2006).

MicroRNAs (miRs) are endogenous, short, non-coding RNAs. Mature miRs are single-stranded RNA molecules of ∼20-25 nucleotides that act as important post-transcriptional regulators of gene expression by binding with their target mRNAs (Singer et al., 2005; Tan et al., 2013). Several miRs have been shown to modulate both neuronal and immune processes (here called NeurimmiRs; Soreq and Wolf, 2011). Recent findings have demonstrated their important roles in neuroinflammation. For example, in prion disease, a uniquely infectious neurodegenerative condition, miR-146a over-expression has been reported in the brain of prion infected mice concurrent with the onset of prion deposition and appearance of activated microglia (Saba et al., 2012). Additionally, miR-146a has also been shown to be induced in response to inflammatory cues, such as IL-1β, as a negative-feedback regulator of the human astrocyte-mediated inflammatory response (Iyer et al., 2012). In Alzheimer’s disease (AD), in which neuroinflammation is a central component, up-regulation of miR-125b was found in the hippocampus and medial frontal gyrus of AD patient (Cogswell et al., 2008). In addition, miR-125b has been reported to repress the expressions of complement factor-H protein (CFH) and interferon regulatory factor 4 (IRF4), which are factors involved in the innate immune system and in the proinflammatory response, in human primary astroglial cells (Lukiw et al., 2012). Moreover, lower levels of miR-181c were found in AD brains, and miR-181c was also down-regulated in the serum of probable AD and mild cognitive impairment (MCI) patients (Geekiyanage et al., 2012). Recent studies have also demonstrated the important roles of miR-181c in the response of astrocytes to inflammatory settings. Over-expression of miR-181c enhanced the LPS-induced increase in interleukin-10 (IL-10) levels, while knockdown of miR-181c resulted in a significant increase in the expression of LPS-induced production of proinflammatory cytokines [IL-1β, TNF-α, interleukin-6 (IL-6), interleukin-8 (IL-8)] and high mobility group box-1 protein (HMGB1) in cultured cortical astrocytes (Hutchison et al., 2013). However, whether these NeurimmiRs are associated with POD remains unknown.

Therefore, the objective of this pilot study was to assess whether the preoperative expression levels of miR-146a, miR-125b, and miR-181c in cerebrospinal fluid (CSF) and serum were associated with POD. We hypothesized that preoperative expression levels of these NeurimmiRs in CSF and serum would be associated with the development and severity of POD. The findings of this investigation may be helpful to create a correlation between the NeurimmiRs expression levels and development of POD, which would promote more studies to investigate the role of NeurimmiRs in the neuropathogenesis of POD and facilitate more miRs biomarker studies of POD.

## Methods

This study was performed in accordance with the Declaration of Helsinki and was approved by the research ethics committee of Zhongnan Hospital of Wuhan University. With written informed consent, we performed a prospective, observational study, which was registered at ClinicalTrials.gov (NCT02817386).

### Study Population

**FIGURE 1:**
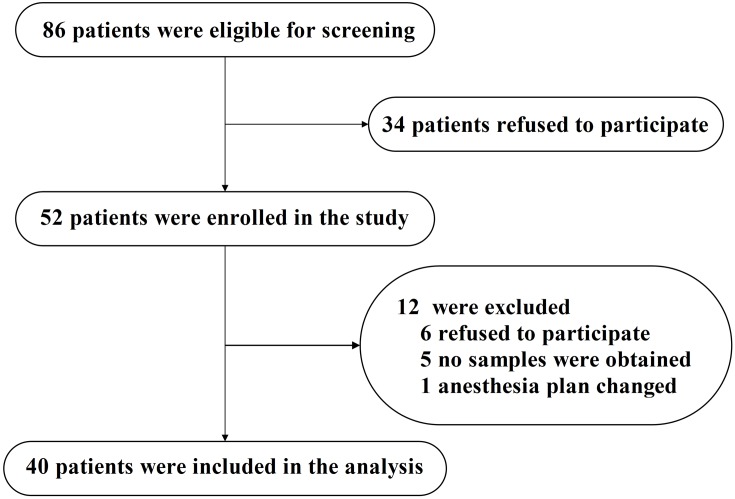
Flow diagram. The flow diagram shows that 86 patients were initially screened for the studies, and 40 patients were finally included in the data analysis.

The study was conducted at Zhongnan Hospital of Wuhan University (Wuhan, China) between March 2015 and February 2016. Eligible patients were at least 65 years old and were scheduled to have total hip/knee replacement under spinal anesthesia. A total of 86 adults were asked to participate in this study (see **Figure 1**, flow diagram). After reviewing patient medical records, patients were excluded if they had (1) a past medical history of neurological or clinically evident neurovascular disease (e.g., AD, other forms of dementia, stroke); (2) inability to read or severe visual or auditory deficits; (3) Mini-Mental State Examination (MMSE) scores of 26 or less; (4) American Society of Anesthesiologists (ASA) score [a global score that assesses the physical status of patients before surgery, ranging from 1 (normal health) to 5 (moribund) (Davenport et al., 2006)] greater than 3; (5) a history of alcohol abuse and drug dependence; or (6) unwillingness to comply with the protocol or procedures.

### Neuropsychological Testing

Each patient was interviewed preoperatively on the first and second postoperative days. The MMSE was administered one day before the scheduled surgery. The assessment of delirium was performed on the first and second days after surgery between 8:00 am and 10:00 am. A visual analog scale (VAS) score of 0–10 (lower score indicating lower level of pain; Chung et al., 2016) was used to assess pain in the patients at the same time. The presence or absence of POD was defined according to the Confusion Assessment Method (CAM), and the severity of POD was defined according to the Memorial Delirium Assessment Scale (MDAS) (Inouye et al., 1990; Schuurmans et al., 2003). The Chinese version of the CAM and MDAS have been proved to have good reliability and validity with use in the Chinese elderly population (Leung et al., 2008; Shi et al., 2014). In this study, the highest CAM and MDAS scores from the postoperative day 1 and day 2 were presented. MDAS scores were evaluated for each patient, regardless of whether he or she met the CAM criteria on that particular day.

### Anesthesia and Surgery

All the participants underwent total hip or total knee replacement under spinal anesthesia by the same surgery team to avoid potential confounding factors owing to varying surgery skills or different surgical practices. Electrocardiography, pulse oximetry and non-invasive blood pressure were continuously monitored during anesthesia and were recorded at fixed intervals of 5 min. 37 patients received propofol during the surgery for sedation. The postoperative pain control included standard postoperative pain management, and postoperative analgesia was restricted to non-opioids (flurbiprofen axetil) unless clinically indicated. All the details of clinical care were documented in case report forms.

### Sample Collection

Peripheral venous blood samples (5 ml) were collected in additive-free vacuum blood tubes from the enrolled patients before anesthesia. Then, serum specimens were isolated by two steps of centrifugation. The blood samples were centrifuged at 3000 *g* for 10 min in 4°C for the collection of supernatants, followed by centrifuging again at 12,000 *g* for 10 min in 4°C for the collection of pure serum (Tan et al., 2014), which was stored at -80°C until further analysis.

The CSF (4 ml) was collected in an RNase-free Eppendorf tube during spinal anesthesia prior to administration of the local anesthetic. The samples were centrifuged immediately at 3000 rpm at 4°C for 10 min to remove cells (Muller et al., 2014), and only the supernatant was retained at -80° until further analysis.

### RNA Extraction, Reverse Transcription, and qRT-PCR

**FIGURE 2:**
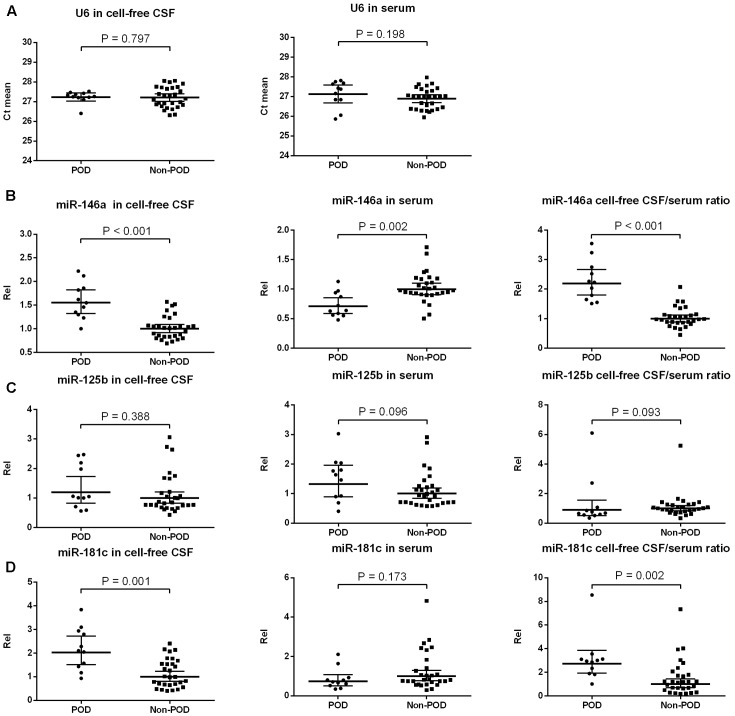
Expression of candidate NeurimmiRs in the CSF and serum of patients with or without POD. Scatter plots of mean Ct-values of U6 **(A)** and expression levels of miR-146a **(B)**, miR-125b **(C)**, and miR-181c **(D)** in the CSF and serum of patients with or without POD. Rel was calculated after normalization using U6 snRNA. The black horizontal lines represent median Rel values ± 95% CIs. Each point represents the mean of triplicate samples. *P*-values were determined using the Mann–Whitney *U*-test. POD, postoperative delirium; Rel, relative expression level; CSF, cerebrospinal fluid.

We extracted total RNA from 2 ml of CSF and 300 μl of serum using TRIzol reagent (Invitrogen, CA, USA) in accordance with the manufacturer’s protocol. The RNA concentration and purity were detected using a NanoDrop ND-1000 spectrophotometer (NanoDrop Technologies, Wilmington, USA) (Supplementary Table S3). Our RNA sample purity was between 1.8 and 2.0. At least 600 ng of total RNA of serum or 60 ng for CSF samples was used to perform reverse transcription using stem-loop RT primers for three candidates (miR-146a, miR-125b, miR-181c) and for endogenous control (U6) according to the manufacturer’s protocol. U6, which provided the most stabilized expression in both the POD group and the non-POD group samples (**Figure 2A**), was used as an endogenous control for the validation of all the selected candidate miRs. The primer sequences were obtained from Invitrogen Biotechnology Co., Ltd (Shanghai, China) and are listed in **Table 1**. In the preliminary experiment, the single peak was observed in each melting curve for candidate miRs and U6 snRNA, which indicate good primer specificity. Quantitative real-time PCR (qRT-PCR) was performed using a StepOne real-time PCR system (Life technologies, CA, USA) with the SYBR Premix Ex Taq (TaKaRa, Dalian, China) according to the manufacturers’ instructions. Each sample was examined in triplicate, and the amounts of the PCR products produced were normalized to the internal control U6. miR expression levels were obtained by relative quantification using the 2^-ΔΔCt^ method. Findings greater or less than 1 were considered to indicate overexpression or underexpression, respectively. All the values were standardized relative to the non-POD values, which were represented as a value of 1.

**Table 1:** Primers sequences for qRT-PCR.

Category	Sequences
miR-146a	RT: 5′-CTCAACTGGTGTCGTGGAGTCGGCAATTCAGTTGAGAACC CATG-3′
	Forward: 5′-CCTGAGAAGTGAATTCCATGGG-3′
	Reverse: 5′-TGGTGTCGTGGAGTCG-3′
miR-125b	RT: 5′-CTCAACTGGTGTCGTGGAGTCGGCAATTCAGTTGAGTCACAAGT-3′
	Forward: 5′-CTTCCCTGAGACCCTAACTTGTG-3′
	Reverse: 5′-TGGTGTCGTGGAGTCG-3′
miR-181c	RT: 5′-CTCAACTGGTGTCGTGGAGTCGGCAATTCAGTTGAGACTCACCG-3′
	Forward: 5′-GAACATTCAACCTGTCGGTGAG-3′
	Reverse: 5′-TGGTGTCGTGGAGTCG-3′
U6	Forward: 5′-CTCGCTTCGGCAGCACAT-3′
	Reverse: 5′-AACGCTTCACGAATTTGCGT-3′

### Statistical Analysis

The data are expressed as the mean ± standard deviation (SD), the median and interquartile range (IQR, 25–75 percentile) or a number (%). POD incidence is presented as a percentage. The Kolmogorov–Smirnov method was used first to test the normality of all the variables. The Mann–Whitney *U*-test was used to analyze the MDAS, VAS, and MMSE scores between the participants who developed POD and those who did not and to determine the differences in CSF and/or serum NeurimmiRs levels between the delirious and non-delirious patients. The Mann–Whitney *U*-test was also used to analyze the differences in MDAS scores between the participants in the first (lowest) quartile of NeurimmiRs levels and the combination of the participants in the second, third, and fourth (highest) quartiles of miR levels. Finally, we applied simple linear regression to determine the associations between the NeurimmiRs levels in CSF and/or serum and MDAS scores, and we used multiple linear regression to determine the associations after adjustment for age, sex and total RNA concentration. The regression coefficient ± standard error (SE) was used to illustrate the association between NeurimmiRs levels and MDAS scores.

Because this was a pilot study, no power calculations were performed; instead we aimed to recruit the maximum number of patients possible with the resources available, which we estimated to be 40. Statistical significance was set at *P* < 0.05. SPSS statistical software, version 21.0 (SPSS, Inc, Chicago, IL, USA), and GraphPad Prism software, version 6.01 (GraphPad Software, Inc, La Jolla, CA, USA), were used for data analysis.

## Results

### Participant Characteristics

Eighty-six eligible participants were screened, 52 of whom provided informed consent for the study. Twelve participants were excluded from the study. The reasons for dropouts are shown in **Figure 1**. Therefore, 40 patients (*n* = 40) remained for analysis. The demographic and clinical data of the participants are summarized in **Table 2**, and the characteristics of the excluded participants are shown in Supplementary Table S1.

**Table 2:** Characteristics of participants.

	*N* = 40
Age (year), mean ± SD	73.8 ± 5.9
Male, *n* (%)	18 (45.0)
Years of education, *n* (%)	
0	9 (22.5)
1–9	19 (47.5)
10–13	7 (17.5)
14–17	2 (5.0)
>17	3 (7.5)
Height (cm), mean ± SD	162.4 ± 7.1
Body weight (kg), mean ± SD	61.3 ± 8.3
BMI (kg/m^2^), mean ± SD	23.2 ± 2.4
ASA class, *n* (%)	
I	0
II	26 (65.0)
III	14 (35.0)
Time of anesthesia (min), mean ± SD	141.5 ± 17.9
Time of surgery (min), mean ± SD	105.6 ± 17.1
Type of surgery	
Total hip arthroplasty/replacement, *n* (%)	17 (42.5)
Total knee arthroplasty/replacement, *n* (%)	23 (57.5)
Estimated blood loss (ml), median and 25–75 percentile	150 (103–200)
Postoperative the highest CAM score, median and 25–75 percentile	18 (16–23)
Postoperative the highest MDAS score, median and 25–75 percentile	7 (5–11)
Postoperative the highest VAS score, median and 25–75 percentile	3 (1–4)

*The length of anesthesia was defined from the time that the anesthesiologists started the spinal anesthesia in the patients to the time when the patients were sent to the post-anesthesia care unit. The length of surgery was defined from the time of initial incision to the time of the closure of the skin. POD, postoperative delirium; ASA, American Society of Anesthesiologists; cm, centimeter; min, minute; kg, kilogram; ml, milliliter; SD, standard deviation; CSF, cerebrospinal fluid.*

The incidence of POD observed at either of the two postoperative assessments was 27.5% (*n* = 11 of the 40 patients). Five of 11 patients who developed POD were male. The median of the highest MDAS score of delirious patients was 13 (11–18) (median and 25–75 percentile), which was higher than that of the non-delirious patients [6 (4–7), *P* < 0.001]. The median of the highest MDAS score of all the participants over the first two postoperative days was 7 (5–11). Because Marcantonio et al. (2002) reported that MDAS scores had prognostic significance even in patients without delirium, we assessed the MDAS scores in the entire population and not only in those who developed POD.

Postoperatively, the highest VAS scores did not differ between patients with delirium 2 (1–3) and without delirium [3 (2–4), *P* = 0.200]. For patients who subsequently developed POD, the preoperative MMSE score [27.5 (27–28)] was not significantly different from the score those who did not develop delirium [28.1 (27–29), *P* = 0.086].

Thirty-seven patients received propofol during the operation for sedation. The mean dose of propofol for patients who developed POD (268.6 ± 33.4 mg) was not significantly different from those who did not develop POD (278.2 ± 34.4 mg, *P* = 0.432).

### Preoperative NeurimmiRs Levels in CSF and/or Serum and POD

U6 has been used before as an internal control in CSF (Gallego et al., 2012) and serum (Zuberi et al., 2016), and it was equally present in POD and non-POD samples with a small variation across the samples in each group (**Figure 2A**). Therefore, it was used for normalization purposes in the current study. We compared the preoperative levels of three candidate NeurimmiRs (miR-146a, miR-125b, and miR-181c) in CSF and/or serum in the participants with POD and those without it. The Mann–Whitney test showed that patients with delirium had a higher CSF miR-146a level, a lower serum miR-146a level, and a higher CSF/serum miR-146a ratio compared to non-delirious patients (**Figure 2B**). However, no significant difference was observed between patients with or without delirium with regard to miR-125b levels in CSF and serum (**Figure 2C**). Additionally, for patients who ultimately developed POD, the CSF level of miR-181c, as well as the ratio of CSF/serum miR-181c, was significantly increased compared to patients who did not develop delirium (**Figure 2D**). However, patients with and without POD did not differ statistically with regard to the serum levels of miR-181c (**Figure 2D**).

### Preoperative NeurimmiRs Levels in CSF and/or Serum and POD Severity

**FIGURE 3:**
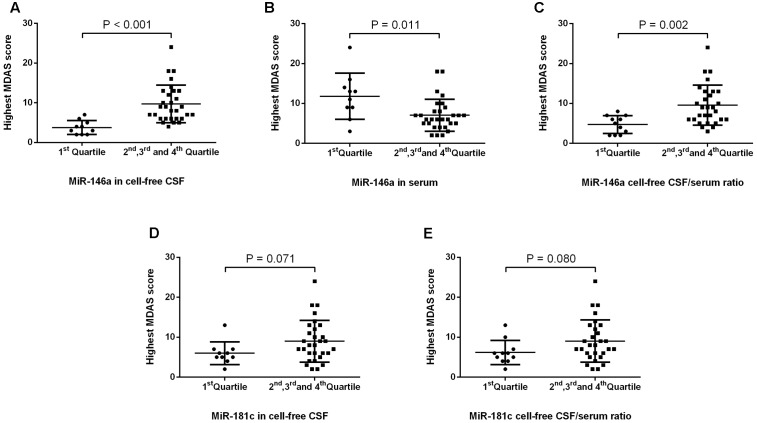
POD severity in the first quartile and the combination of the other three quartiles of levels of miRs and miR CSF/serum ratios. We found that the median of the highest MDAS score (4, 2–5) of the patients in the first quartile of miRNA-146a in CSF was significantly lower than that of the patients in the combination of the second, third, and fourth quartiles of miRNA-146a in CSF (9, 6–13) **(A)**. In contrast, the median of the highest MDAS score (12, 8–14) of the patients in the first quartile of miRNA-146a in serum was significantly higher than that of the patients in the combination of the second, third, and fourth quartiles of miRNA-146a in serum (6, 5–8) **(B)**. The median of the highest MDAS score in the first quartile of miR-146a cell-free CSF/serum ratio (5, 2–7) was significantly lower than that of the patients in the combination of the second, third, and fourth quartiles of miR-146a CSF/serum ratio (9, 6–13) **(C)**. No significant difference in the highest MDAS score was observed between the patients in the first quartile and the patients in the combination of the second, third, and fourth quartiles of CSF miR-181c or CSF/serum miR-181c ratio **(D,E)**. The black horizontal lines represent median MDAS scores ± interquartile ranges. Each point represents the highest MDAS score. *P*-values were determined using the Mann–Whitney *U*-test. POD, postoperative delirium; CSF, cerebrospinal fluid; MDAS, Memorial Delirium Assessment Scale.

Next, we investigated whether the levels of miR-146a and miR-181c in CSF and serum, as well as the ratios of CSF/serum miR-146a and miR-181c, were associated with POD severity. MDAS has been used to assess the severity of delirium symptoms based on 10 features (Shi et al., 2014). In the current study, MDAS scores were evaluated for each patient, regardless of whether he or she met the CAM criteria, given that MDAS scores had prognostic significance even in patients without delirium (Marcantonio et al., 2002). We first compared the MDAS score, the measurement of delirium severity, between the patients in the first quartile and the patients in the combination of the second, third, and fourth quartiles. We found that the patients in the lowest quartile of CSF miR-146a had lower MDAS scores (**Figure 3A**) than those of the patients in the combination of the second, third, and fourth quartiles of CSF miR-146a. Similarly, the highest MDAS score of the patients in the first quartile of CSF/serum miR-146a ratio was lower than that of the patients in the combination of the second, third, and fourth quartiles of CSF/serum miR-146a ratio (**Figure 3C**). Conversely, the highest MDAS score of the patients in the first quartile of serum miR-146a was higher than that of the patients in the combination of the second, third, and fourth quartiles of serum miR-146a (**Figure 3B**). However, no significant difference in the highest MDAS score was observed between the patients in the first quartile and the patients in the combination of the second, third, and fourth quartiles of CSF miR-181c or CSF/serum miR-181c ratio (**Figures 3D,E**).

Then, we assessed the relationship between the NeurimmiRs levels in CSF and/or serum and MDAS scores. The data were fit by linear regression analysis (**Supplementary Figure S1**) and using unadjusted simple linear regression, we found that the preoperative CSF level of miR-146a, as well as the ratios of CSF/serum miR-146a and miR-181c, were significantly correlated (positively) with the highest MDAS score. However, the preoperative serum level of miR-146a was significantly correlated (negatively) with the highest MDAS score (**Table 3**). Multiple linear regression, after adjusting for age, sex, and total RNA concentration, showed that the preoperative CSF miR-146a level, as well as the ratios of CSF/serum miR-146a and miR-181c, remained significantly correlated (positively) with the highest MDAS score (**Table 3**).

**Table 3:** Correlation between MDAS score and the levels of miRs or ratios.

Highest MDAS score	Unadjusted		Adjusted by age, sex, and total RNA concentration	
	Regression coefficient ± SE	*P*-value	Regression coefficient ± SE	*P*-value
miR-146a in CSF	0.852 ± 1.087	<0.001^∗∗∗^	0.872 ± 1.101	<0.001^∗∗∗^
miR-146a in serum	-0.400 ± 2.659	0.011^∗∗^	-0.403 ± 2.750	0.026^∗^
miR-146a CSF/serum ratio	0.817 ± 0.643	<0.001^∗∗∗^	0.817 ± 0.652	<0.001^∗∗∗^
miR-181c in CSF	0.680 ± 0.698	<0.001^∗∗∗^	0.682 ± 0.720	<0.001^∗∗∗^
miR-181c CSF/serum ratio	0.307 ± 0.424	0.054	0.302 ± 0.439	0.078

*The left panel of the table illustrates the results of the regression coefficients for miR-146a in CSF, miR-146a in serum, miR-146a CSF/serum ratio, miR-181c in CSF and miR-181c CSF/serum ratio with the highest MDAS score in a simple linear regression. The right panel of the table shows the regression coefficients in multiple linear regressions, including adjustment for confounding factors. MDAS, Memorial Delirium Assessment Scale; CSF, cerebrospinal fluid; SE, standard error, ^∗^P < 0.05; ^∗∗^P < 0.01; ^∗∗∗^P < 0.001.*

## Discussion

In this pilot study, we assessed the associations between preoperative expressions of NeurimmiRs (miR-146a, miR-125b, and miR-181c) in CSF and serum and POD in 52 older adults who underwent total hip and knee replacement under spinal anesthesia. We found up-regulation of miR-146a and miR-181c in CSF and down-regulation of miR-146a in the serum of patients who developed POD. Additionally, the delirious patients had higher CSF/serum ratios of miR-146a and miR-181c levels than the non-delirious patients. We also found that lower CSF miR-146a and CSF/serum miR-146a ratios were significantly associated with milder POD severity, as represented by a lower MDAS score. Taken together, these findings suggested that miR-146a and miR-181c might participate in the neuropathogenesis of POD, pending further investigation.

Previous studies have demonstrated that miRs are stably expressed in various body fluids (Weber et al., 2010), and their unique expression patterns can serve as fingerprints of neurological diseases such as AD (Denk et al., 2015). CSF is in direct contact with the extracellular space of the brain, and it can reflect the biochemical changes that occur in the brain. Therefore, it is the optimal source of POD biomarkers (Xie et al., 2014). Serum is less invasive and more readily available, and circulating miRs are attractive candidates for monitoring central nervous system (CNS) diseases such as AD (Tan et al., 2014). Accordingly, in the present study, we measured the expression levels of miRs in both CSF and serum in patients who underwent total hip and knee replacement under spinal anesthesia. In addition, the CSF/serum ratio has been reported to a useful parameter for the early diagnosis of CNS diseases (Mitchell et al., 2008; Mikecin et al., 2013; Schmidt et al., 2015), and it could exclude the influence of the physiology and pathology of the participants (Mikecin et al., 2013). Thus, we also calculated the CSF/serum ratios of miRs levels in this study.

The incidence of POD following total joint replacement in our study (27.5%) was within the reported range of 3.6–41% (Rudolph and Marcantonio, 2011; Xie et al., 2014; Scott et al., 2015). For example, Xie et al. (2014) reported that POD occurred in 20% of patients (≥63 years old) who underwent total hip and knee replacement under spinal anesthesia. The variation in the POD incidence could be due to age and the influence of perioperative factors, such as postoperative pain (Leung et al., 2013) and sleep disturbances (Leung et al., 2015). Therefore, the incidence of POD (27.5%) in this study demonstrated the validity of our delirium assessment methods. However, in this pilot study, the high dropout rate and small sample size might have resulted in consequent uncertainty about the incidence, and this requires further investigation.

Despite an increase of studies focused on the identifications of POD risk factors, the POD molecular mechanisms are still largely unknown (Maclullich et al., 2008) and notably, the possibility for early identification of patients who may develop POD is still to be better defined. The neurotransmitters imbalance hypothesis (Alagiakrishnan and Wiens, 2004) and inflammatory hypothesis (Hala, 2007; Cerejeira et al., 2010; van Gool et al., 2010) are the most widely propagated theories for POD neuropathogenesis. POD has been suggested to be related to neuroinflammation (Cerejeira et al., 2010; van Gool et al., 2010), and it is a marker of brain vulnerability. Its occurrence suggests the possibility of underlying neurological disease, such as early or preclinical dementia (Marcantonio et al., 1994; Inouye and Ferrucci, 2006; Davis et al., 2012). Surgery and anesthesia might unmask the underlying pathology (Gunther et al., 2012). In other words, major surgery and anesthesia could be considered a stress test for the brain, with POD as a “positive” result of the test, revealing brain vulnerability. Thus, it is plausible that patients who develop POD have some special underlying changes in the vulnerable brain that facilitate neuroinflammation induced by surgery and anesthesia. In our study, the preoperative changes in miR-146a and miR-181c levels in CSF and serum in patients who developed POD indicated that the deregulation of these NeurimmiRs could be one of these changes in the vulnerable brain.

Increasing evidence has demonstrated the involvement of miR-146a in the regulation of inflammation in human neurological disorders. In physiological conditions, transcription of miR-146a occurs at baseline levels; however, initiation of proinflammatory Toll-like receptor (TLR) signaling immediately results in strong co-induction of its expression through a mechanism that is largely NF-κB-dependent (Boldin and Baltimore, 2012). In addition, miR-146a could also be induced as a negative-feedback regulator of the astrocyte-mediated inflammatory response (Iyer et al., 2012). Furthermore, in AD patients, miR-146a has been shown to down-regulate CFH, which is an important repressor of innate immunity acting on the cerebral inflammation response (Lukiw and Alexandrov, 2012; Lukiw et al., 2012). Similarly, miR-181c plays important roles in the response to inflammatory settings. It negatively regulates the production of multiple cytokines and fibroblast growth factor 2 (FGF2) in astrocytes, effectively suppressing their production in an inflammatory environment (Hutchison et al., 2013). miR-181c can also directly regulate TNF-α and TLR4 production post-transcriptionally, inhibiting NF-κB-mediated inflammation (Zhang et al., 2012, 2015; Hutchison et al., 2013). Collectively, these NeurimmiRs (miR-146a and miR-181c) have been implicated in the regulation of the immune and inflammatory responses. It is plausible that their abnormal expression in the CSF and serum of patients with POD might reflect a low-level chronic inflammatory state in the vulnerable brain before surgery. Surgical procedures can trigger systemic inflammation (Dantzer et al., 2008; Cerejeira et al., 2010; MacLullich et al., 2011), exaggerate neuroinflammation in the primed vulnerable brain and lead to delirium (Murray et al., 2012). Aging and neurodegenerative diseases are well-known predisposing risk factors for delirium (Inouye and Charpentier, 1996), and they are also accompanied by a low-level chronic inflammatory state (Olivieri et al., 2013). Bhaumik et al. (2009) found that some miRs, for example, miR-146a expression significantly increased during senescence of human fibroblasts. In our study, we focused on patients who were at least 65 years old (mean age: 73.8 ± 5.9 years). Therefore, it is possible that the presence of miR-146a in patients without POD was aging based.

In our study, the up-regulation of miR-146a and miR-181c in CSF and the down-regulation of miR-146a in serum were observed in patients who developed POD, while no difference was observed between patients with or without POD with regard to miR-125b levels in CSF and serum. Up-regulation of miR-146a has been reported in the brains of human beings and in mouse models of prion disease (Saba et al., 2008), consistent with our results. However, miR changes in POD have not always been consistent among studies of neuroinflammation-related diseases. For example, in contrast to our observations, recent studies have reported reduced levels of miR-146a and miR-125b in both the serum and CSF of AD patients (Kiko et al., 2014; Muller et al., 2014) and reduced miR-181c in the serum of probable AD and MCI patients (Geekiyanage et al., 2012). Such discrepancies could originate from study-related parameters, such as variations in sample size, type of disease and patient profiles. Interestingly, in our studies, miR-146a and miR-181c expression levels differed between CSF and serum. This outcome was not surprising, given that the miRs in CSF are derived from neural cells, whereas serum miRs are collected from all of the tissues in the body (Burgos et al., 2013). Thus, do serum miRs have crosstalk with CSF miRs? It is unlikely that miRs alone can cross the BBB (Burgos et al., 2013). However, it has been reported that exosomes, which are secreted from cells (Street et al., 2012; Raposo and Stoorvogel, 2013) and facilitate intercellular communication by transporting molecules, such as miRs (Duijvesz et al., 2011; Kharaziha et al., 2012; Hannafon and Ding, 2013), can cross the BBB (Alvarez-Erviti et al., 2011). The study by Yagi et al. (2017) demonstrated that miR enrichment in the exosomal fractions relative to the non-exosomal fractions of both CSF and serum and miR expression profiles in exosome fractions differed between CSF and serum. In particular, the dominantly expressed exosomal miRs were very different between CSF and serum. These results suggested that the brain is a major source of CSF exosomal miRs, although a small fraction of CSF exosomal miRs absent from the brain might have been derived from leucocytes in CSF (Gombar et al., 2012). Importantly, these observations also suggested that exosomal miR translocation between the blood and CSF could be rare. However, the communication of exosomal miRs between CSF and serum could be affected and could vary under pathological conditions, especially in diseases affecting BBB function (Yagi et al., 2017). However, to date, the causal relationship between BBB permeability and POD remains uncertain, although Acharya et al. (2015) reported that inhaled anesthetics, such as sevoflurane and isoflurane, act directly on brain vascular endothelial cells to increase BBB permeability, thereby contributing to POD. Therefore, future studies to investigate the communication between miRs in CSF and serum are warranted.

In comparison with sevoflurane anesthesia, propofol anesthesia has been shown to be associated with a lower incidence (6.9%) of POD in elderly patients (Ishii et al., 2016). In the current study, the mean dose of propofol for patients who developed POD was not significantly different from those who did not develop POD. Therefore, these results suggest that propofol sedation may not significantly affect the development of POD, pending further investigations.

In the current study, we not only applied the MMSE to exclude participants with dementia, but we also compared differences in the MMSE at baseline between the participants with or without POD. We found that, for patients who subsequently developed POD, the preoperative MMSE score was not significantly different from that of subjects who did not develop delirium. However, it is known that prior cognitive impairment is a major risk factor for delirium (Davis et al., 2015). Moreover, delirium risk in the elderly population is indirectly proportional to baseline MMSE score. For instance, the probability of incident delirium at follow-up for an 85-year-old man with MMSE = 28 points at baseline would be 0.12, increasing to 0.29 for an equivalent individual with an MMSE score of 10 points at baseline (Davis et al., 2015). These discrepancies could be explained by issues of the small sample size and the ceiling effects of the MMSE (Xu et al., 2002).

There were some potential limitations of our study. First, the current pilot study was not sufficiently powered to provide strong evidence for the role of NeurimmiRs in POD due to the small sample size and high dropout rate. A larger study with adequate power is indicated to validate our results. Second, we removed the cells from the CSF according to the methods as described by Muller et al. (2014), considering that the influence of blood contamination on CSF miR levels is a potential confounding factor. However, the presence of blood cells in CSF, even if they are removed using centrifugation before analysis, can lead to bias in the expression levels of miRs (Muller et al., 2014). Therefore, to avoid any bias in the results, we should determine the number of blood cells in CSF samples when measuring miR levels in future studies. Third, we measured delirium only once daily; given the fluctuating nature of delirium, we might have underestimated its incidence. Fourth, only one internal control (snRNA U6) has been used in the current study. We should use more controls in the further investigations. Finally, NeurimmiRs play a significant role in the pathophysiological context, especially in the neuroinflammatory process and neurodegenerative diseases (Supplementary Table S2). In this study, we just focused on three of them: miR-146a, miR-125b, and miR-181c. However, there might be more NeurimmiRs, such as miR-124 and miR-132, that could contribute to neuroinflammation (Soreq and Wolf, 2011) and the neuropathogenesis of POD. We propose that the specific serum and CSF miR expression profiles [not a single miR or a few miR(s)] constitute the fingerprint of POD, which could have an enormous impact on diagnosis and personalized medicine in the future.

In summary, dysregulation of preoperative miR-146a and miR-181c in CSF and serum was associated with the development and severity of POD. To date, it remains unknown whether there is a causal relationship or an association between these NeurimmiRs expression levels and the development of POD. Hence, further studies are required to clarify this question. These studies will also hopefully facilitate miRs biomarker studies of POD.

## Author Contributions

RD contributed to study design, data collection, statistical analysis, and manuscript preparation. LS performed qRT-PCR. YL involved in data collection. XY performed neuropsychological testing. MP contributed to study concept and design, manuscript preparation and review. ZZ performed statistical analysis.

## Conflict of Interest Statement

The authors declare that the research was conducted in the absence of any commercial or financial relationships that could be construed as a potential conflict of interest.

## Supplementary Material

FIGURE S1 CSF miR-146a expression level and MDAS scores data test results.
