# Supplementary figures and images for: NeurimmiRs and Postoperative Delirium in Elderly Patients Undergoing Total Hip/Knee Replacement: A Pilot Study
Source: Front Aging Neurosci. 2017 Jun 23;9:200. doi: 10.3389/fnagi.2017.00200 (PMC5481321; doi:10.3389/fnagi.2017.00200)

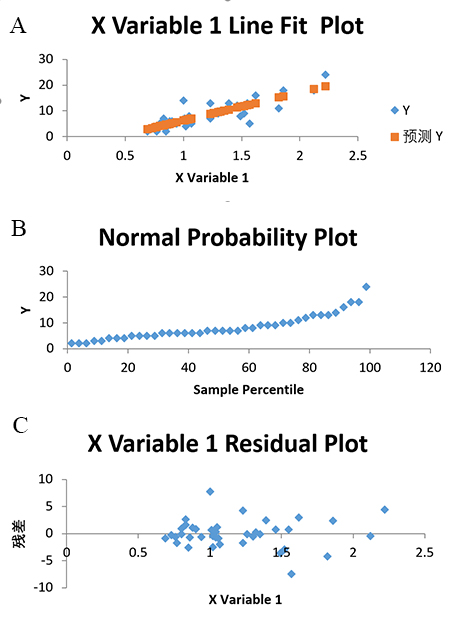

Supplement: FIGURE S1 — CSF miR-146a expression level and MDAS scores data test results. [file Image_1.JPEG]
