# Supplementary material for: NeurimmiRs and Postoperative Delirium in Elderly Patients Undergoing Total Hip/Knee Replacement: A Pilot Study
Source: Front Aging Neurosci. 2017 Jun 23;9:200. doi: 10.3389/fnagi.2017.00200 (PMC5481321; doi:10.3389/fnagi.2017.00200)
Supplement: Supplementary file 2 [file Table_1.DOCX]

Supplemental Table 1 Characteristics of the excluded participants

| Sample | The reason for the excluded | Age | Gender | Years of education | Height (cm) | Body weight (kg) | ASA | Time of anesthesia(min) | Time of surgery(min) | Type of surgery | Estimated blood loss | Preoperative MMSE score |
| --- | --- | --- | --- | --- | --- | --- | --- | --- | --- | --- | --- | --- |
| 1 | Anesthesia plan changed | 65 | Female | 12 | 155 | 50 | Ⅱ | *null* | *null* | *null* | *null* | 27 |
| 2 | No samples were obtained | 81 | Male | 15 | 178 | 60 | Ⅲ | *null* | *null* | *null* | *null* | 27 |
| 3 |  | 74 | Female | 16 | 158 | 51 | Ⅲ | *null* | *null* | *null* | *null* | 28 |
| 4 |  | 88 | Male | 9 | 175 | 55 | Ⅱ | *null* | *null* | *null* | *null* | 29 |
| 5 |  | 76 | Male | 9 | 168 | 60 | Ⅱ | *null* | *null* | *null* | *null* | 28 |
| 6 |  | 75 | Female | 5 | 156 | 40 | Ⅲ | *null* | *null* | *null* | *null* | 29 |
| 7 | Patients or family members refused to participate the study after surgery | 79 | Female | 9 | 154 | 45 | Ⅲ | 150 | 120 | Hip | 350 | 29 |
| 8 |  | 66 | Female | 5 | 154 | 43 | Ⅲ | 120 | 100 | Hip | 300 | 28 |
| 9 |  | 75 | Female | 5 | 160 | 52 | Ⅱ | 170 | 145 | Knee | 160 | 27 |
| 10 |  | 75 | Male | 9 | 163 | 72 | Ⅲ | 185 | 125 | Hip | 300 | 29 |
| 11 |  | 68 | Female | 16 | 158 | 55 | Ⅱ | 150 | 120 | Hip | 300 | 30 |
| 12 |  | 77 | Male | 12 | 170 | 74 | Ⅲ | 200 | 160 | Knee | 450 | 28 |

ASA, American Society of Anesthesiologists
