# Supplementary material for: NeurimmiRs and Postoperative Delirium in Elderly Patients Undergoing Total Hip/Knee Replacement: A Pilot Study
Source: Front Aging Neurosci. 2017 Jun 23;9:200. doi: 10.3389/fnagi.2017.00200 (PMC5481321; doi:10.3389/fnagi.2017.00200)
Supplement: Supplementary file 3 [file Table_2.DOCX]

Supplemental Table 2 Summary on target genes of miR-146a, miR-125b and miR-181c

| MiRNAs | Target Gene | Organ, Tissues or Cells Expressed | Disease Expressed | Roles of miRNAs Or Potentials of Clinical Implications | References |
| --- | --- | --- | --- | --- | --- |
| MiR-146a | IRAK1, TRAF6, CFH, EGFR, CARD10, STAT1, WASF2，ROCK1, SOD2, PTGES-2, L1CAM，TSPAN12…… | Brain, lung, cerebrospinal fluid, serum/plasma, PBMCs , BMSCs, gastric cancer cells, endothelial cells, dendritic cells, monocytes, neuronal and microglial cells， macrophages…… | Cancer cell invasion and metastasis, neurodegenerative disorders（e.g.AD，MS, age-related macular degeneratio）, autoimmune diseases（e.g.SLE，RA, psoriatic arthritis）…… | Neuroinflammation  A critical regulator of both the innate and adaptive immune systems  Controlled TLR/NF-κB signaling in inflammatory settings | ^[^[^1-12^](#_ENREF_1)^]^ |
| MiR-125b | CFH, IRF4, CDKN2A, SYN-2,15-LOX,TNFAIP3, HOTAIRM1, ULK1, E2F1, DRAM2, CXCL13, CCR5, VPS4B…… | Brain, cerebrospinal fluid , serum/plasma, neuronal-glial cells, microglia, monocytes…… | Neurodegenerative disorders（e.g.AD, ALS）, cardiovascular disease…… | Neuroinflammation.  Activate immune response  Astrogliosis and glial cell proliferation  Synaptic deficits and defective  Neurotrophic deficits  Autophagy | ^[^[^12-20^](#_ENREF_12)^]^ |
| MiR-181c | TGFBI, TRIM2, SIRT1, BTBD3, oncogene RAS, TNF-α,MeCP2, XIAP…… | Brain,cerebrospinal fluid , serum/plasma, neuronal-glial cells , microglia, hematopoiesis…… | Neurodegenerative disorders（e.g.AD, PD）, acute myeloid leukemia…… | Neuroinflammation.  Down-regulated by Amyloid-β  Neuronal apoptosis | ^[^[^21-23^](#_ENREF_21)^]^ |

IRAK1: interleukin-1 receptor-associated kinase 1; TRAF6: TNF receptor-associated factor 6; CFH：complement factor-H; EGFR: epidermal growth factor receptor; WASF2: WASP family verprolin homologous protein 2; ROCK1: Rho-activated protein kinase; SOD2: superoxide dismutase 2; PTGES-2: prostaglandin E2 synthase-2; L1CAM: L1 cell adhesion molecule; TSPAN12: βAPP-membrane associated tetraspanin-12; PBMCs: peripheral blood mononuclear cells; BMSCs: bone marrow stem cells; AD: Alzheimer`s Disease; MS: multiple sclerosis; SLE: systemic lupus erythematosus; RA: Rheumatoid arthritis; IRF4:  interferon regulatory factor 4; CDKN2A: cyclin-dependent kinase inhibitor 2A; SYN-2：synapsin genes-2; 15-LOX: 15-lipoxygenase; TNFAIP3: TNF alpha-induced protein 3; ULK1: unc-51 like autophagy activating kinase 1; E2F1: E2F transcription factor 1; DRAM2: DNA-damage regulated autophagy modulator 2; CXCL13: Chemokine (C-X-C motif) ligand 13; CCR5：Chemokine receptor 5; VPS4B：Vacuolar protein sorting 4 homolog B; ALS: amyotrophic lateral sclerosis; TGFBI：Transforming growth factor beta-induced; TRIM2: Tripartite motif-containing 2; SIRT1: Sirtuin (silent mating type information regulation 2 homolog); BTBD3: BTB domain containing 3; TNF-α：Tumor necrosis factor-α; MeCP2: methyl CpG binding protein 2; XIAP: X-linked inhibitor of apoptosis; PD: Parkinson's disease

References

[1]. Rau CS, Yang JC, Chen YC,et al. Lipopolysaccharide-induced microRNA-146a targets CARD10 and regulates angiogenesis in human umbilical vein endothelial cells[J]. Toxicological sciences : an official journal of the Society of Toxicology,2014,140(2):315-326.

[2]. Wang S, Zhang X, Ju Y,et al. MicroRNA-146a feedback suppresses T cell immune function by targeting Stat1 in patients with chronic hepatitis B[J]. Journal of immunology,2013,191(1):293-301.

[3]. Yao Q, Cao Z, Tu C,et al. MicroRNA-146a acts as a metastasis suppressor in gastric cancer by targeting WASF2[J]. Cancer letters,2013,335(1):219-224.

[4]. Ji G, Lv K, Chen H,et al. MiR-146a regulates SOD2 expression in H2O2 stimulated PC12 cells[J]. PloS one,2013,8(7):e69351.

[5]. Matysiak M, Fortak-Michalska M, Szymanska B,et al. MicroRNA-146a negatively regulates the immunoregulatory activity of bone marrow stem cells by targeting prostaglandin E2 synthase-2[J]. Journal of immunology,2013,190(10):5102-5109.

[6]. Hou Z, Yin H, Chen C,et al. microRNA-146a targets the L1 cell adhesion molecule and suppresses the metastatic potential of gastric cancer[J]. Molecular medicine reports,2012,6(3):501-506.

[7]. Xia P, Fang X, Zhang ZH,et al. Dysregulation of miRNA146a versus IRAK1 induces IL-17 persistence in the psoriatic skin lesions[J]. Immunology letters,2012,148(2):151-162.

[8]. Zeng Z, Gong H, Li Y,et al. Upregulation of miR-146a contributes to the suppression of inflammatory responses in LPS-induced acute lung injury[J]. Experimental lung research,2013,39(7):275-282.

[9]. Falcao AS, Carvalho LA, Lidonio G,et al. Dipeptidyl vinyl sulfone as a novel chemical tool to inhibit HMGB1/NLRP3-inflammasome and inflamma-miRs in Abeta-mediated microglial inflammation[J]. ACS chemical neuroscience,2016,

[10]. Saba R, Sorensen DL, Booth SA. MicroRNA-146a: A Dominant, Negative Regulator of the Innate Immune Response[J]. Frontiers in immunology,2014,5:578.

[11]. Sheppard HM, Verdon D, Brooks AE,et al. MicroRNA regulation in human CD8+ T cell subsets--cytokine exposure alone drives miR-146a expression[J]. Journal of translational medicine,2014,12:292.

[12]. Lukiw WJ, Andreeva TV, Grigorenko AP,et al. Studying micro RNA Function and Dysfunction in Alzheimer's Disease[J]. Frontiers in genetics,2012,3:327.

[13]. Pogue AI, Cui JG, Li YY,et al. Micro RNA-125b (miRNA-125b) function in astrogliosis and glial cell proliferation[J]. Neuroscience letters,2010,476(1):18-22.

[14]. Valtorta F, Pozzi D, Benfenati F,et al. The synapsins: multitask modulators of neuronal development[J]. Seminars in cell & developmental biology,2011,22(4):378-386.

[15]. Evergren E, Benfenati F, Shupliakov O. The synapsin cycle: a view from the synaptic endocytic zone[J]. Journal of neuroscience research,2007,85(12):2648-2656.

[16]. Palacios-Pelaez R, Lukiw WJ, Bazan NG. Omega-3 essential fatty acids modulate initiation and progression of neurodegenerative disease[J]. Molecular neurobiology,2010,41(2-3):367-374.

[17]. Lukiw WJ, Alexandrov PN. Regulation of complement factor H (CFH) by multiple miRNAs in Alzheimer's disease (AD) brain[J]. Molecular neurobiology,2012,46(1):11-19.

[18]. Chen ZH, Wang WT, Huang W,et al. The lncRNA HOTAIRM1 regulates the degradation of PML-RARA oncoprotein and myeloid cell differentiation by enhancing the autophagy pathway[J]. Cell death and differentiation,2016,

[19]. Ninomiya M, Kondo Y, Kimura O,et al. The expression of miR-125b-5p is increased in the serum of patients with chronic hepatitis B infection and inhibits the detection of hepatitis B virus surface antigen[J]. Journal of viral hepatitis,2016,23(5):330-339.

[20]. Parisi C, Napoli G, Amadio S,et al. MicroRNA-125b regulates microglia activation and motor neuron death in ALS[J]. Cell death and differentiation,2016,23(3):531-541.

[21]. Kumar S, Reddy PH. Are circulating microRNAs peripheral biomarkers for Alzheimer's disease?[J]. Biochimica et biophysica acta,2016,1862(9):1617-1627.

[22]. Femminella GD, Ferrara N, Rengo G. The emerging role of microRNAs in Alzheimer's disease[J]. Frontiers in physiology,2015,6:40.

[23]. Hutchison ER, Kawamoto EM, Taub DD,et al. Evidence for miR-181 involvement in neuroinflammatory responses of astrocytes[J]. Glia,2013,61(7):1018-1028.
