# Supplementary material for: NeurimmiRs and Postoperative Delirium in Elderly Patients Undergoing Total Hip/Knee Replacement: A Pilot Study
Source: Front Aging Neurosci. 2017 Jun 23;9:200. doi: 10.3389/fnagi.2017.00200 (PMC5481321; doi:10.3389/fnagi.2017.00200)
Supplement: Supplementary file 4 [file Table_3.DOCX]

Table S3 General information on samples and measurement results

| Sample ID | Group | RNA concentration (ng/μl) | | RNA purity | |
| --- | --- | --- | --- | --- | --- |
|  |  | CSF | serum | CSF | serum |
| 01 | POD | 5.68 | 39.12 | 1.82 | 1.82 |
| 02 |  | 5.72 | 66.36 | 1.83 | 1.84 |
| 03 |  | 3.20 | 37.12 | 1.83 | 1.85 |
| 04 |  | 5.20 | 66.08 | 1.81 | 1.81 |
| 05 |  | 3.12 | 65.24 | 1.81 | 1.86 |
| 06 |  | 6.72 | 52.36 | 1.81 | 1.91 |
| 07 |  | 4.52 | 56.64 | 1.85 | 1.80 |
| 08 |  | 2.92 | 27.68 | 1.87 | 1.85 |
| 09 |  | 5.92 | 40.72 | 1.83 | 1.82 |
| 10 |  | 3.08 | 40.96 | 1.88 | 1.81 |
| 11 |  | 6.32 | 31.76 | 1.86 | 1.81 |
| 12 | Non-POD | 6.76 | 38.44 | 1.82 | 1.81 |
| 13 |  | 4.84 | 64.56 | 1.81 | 1.82 |
| 14 |  | 3.36 | 37.84 | 1.86 | 1.88 |
| 15 |  | 3.56 | 65.92 | 1.82 | 1.82 |
| 16 |  | 6.28 | 38.12 | 1.83 | 1.86 |
| 17 |  | 3.88 | 30.44 | 1.87 | 1.87 |
| 18 |  | 3.68 | 59.08 | 1.80 | 1.83 |
| 19 |  | 5.24 | 50.60 | 1.85 | 1.82 |
| 20 |  | 5.20 | 33.36 | 1.81 | 1.82 |
| 21 |  | 5.24 | 38.12 | 1.85 | 1.84 |
| 22 |  | 6.08 | 58.08 | 1.85 | 1.81 |
| 23 |  | 6.56 | 47.44 | 1.82 | 1.80 |
| 24 |  | 3.44 | 69.32 | 1.83 | 1.80 |
| 25 |  | 2.88 | 55.48 | 1.85 | 1.87 |
| 26 |  | 4.04 | 56.40 | 1.84 | 1.83 |
| 27 |  | 7.24 | 64.44 | 1.89 | 1.84 |
| 28 |  | 5.20 | 38.28 | 1.83 | 1.82 |
| 29 |  | 4.48 | 32.20 | 1.89 | 1.86 |
| 30 |  | 5.24 | 55.72 | 1.90 | 1.86 |
| 31 |  | 4.28 | 47.20 | 1.83 | 1.85 |
| 32 |  | 5.44 | 67.04 | 1.94 | 1.87 |
| 33 |  | 5.28 | 37.44 | 1.83 | 1.81 |
| 34 |  | 5.40 | 35.88 | 1.82 | 1.82 |
| 35 |  | 5.64 | 61.84 | 1.83 | 1.80 |
| 36 |  | 4.84 | 50.04 | 1.81 | 1.86 |
| 37 |  | 5.24 | 45.80 | 1.82 | 1.83 |
| 38 |  | 5.28 | 42.92 | 1.83 | 1.80 |
| 39 |  | 2.96 | 44.40 | 1.80 | 1.81 |
| 40 |  | 4.68 | 62.12 | 1.86 | 1.84 |
